# IR-UWB Pulse Generation Using FPGA Scheme for through Obstacle Human Detection

**DOI:** 10.3390/s20133750

**Published:** 2020-07-04

**Authors:** Lalida Tantiparimongkol, Pattarapong Phasukkit

**Affiliations:** Department of Electronics Engineering, Faculty of Engineering, King Mongkut’s Institute of Technology Ladkrabang, Bangkok 10520, Thailand; 61601146@kmitl.ac.th

**Keywords:** IR-UWB radar, FPGA, delay-line, human through-obstacle detection, range estimation, respiratory rate, doppler frequency

## Abstract

This research proposes a scheme of field programmable gate array (FPGA) to generate an impulse-radio ultra-wideband (IR-UWB) pulse. The FPGA scheme consists of three parts: digital clock manager, four-delay-paths stratagem, and edge combiner. The IR-UWB radar system is designed to detect human subjects from their respiration underneath the rubble in the aftermath of an earthquake and to locate the human subjects based on range estimation. The proposed IR-UWB radar system is experimented with human subjects lying underneath layers of stacked clay bricks in supine and prone position. The results reveal that the IR-UWB radar system achieves a pulse duration of 540 ps with a bandwidth of 2.073 GHz (fractional bandwidth of 1.797). In addition, the IR-UWB technology can detect human subjects underneath the rubble from respiration and identify the location of human subjects by range estimation. The novelty of this research lies in the use of the FPGA scheme to achieve an IR-UWB pulse with a 2.073 GHz (117 MHz–2.19 GHz) bandwidth, thereby rendering the technology suitable for a wide range of applications, in addition to through-obstacle detection.

## 1. Introduction

Radar technology first emerged during World War II [1] and has evolved ever since. Apart from military operations, modern radar technology is deployed in numerous applications, including range/speed detection, autonomous driving vehicles [2,3,4,5], detection of objects buried underground (e.g., landmines or pipelines) [6,7,8,9,10], detection of airborne objects [11,12,13], and bio-radiolocation.

In bio-radiolocation, the Doppler radar principle is utilized to remotely detect human life using phase shift of the reflected signal from physiological movements, such as respiration, heartbeat, limb motion [14,15]. In [14], Doppler frequency was used to detect human life through an obstacle (i.e., through-wall detection). Additionally, the Doppler radar method is adopted for medical diagnostics, including sleep apnea, sudden infant death syndrome, and respiratory muscle weakness [16,17,18,19,20,21].

In through-wall detection, there are three conventional radar technologies: continuous wave (CW), frequency-modulated continuous-wave (FMCW), and step frequency continuous wave (SFCW) radars. In [22,23,24], CW radar technology was employed to detect human vital signs underneath the rubble. However, CW radar technology fails to estimate the range (location) of the human buried underneath and requires very strong vital signs for reliable detection [25]. As a result, FMCW and SFCW radar technologies were proposed to address the drawbacks [26,27,28]. Nevertheless, CW, FMCW, and SFCW radars suffer from limited penetration capability [29].

Ultra-wideband (UWB) radar is an unmodulated radar technology that transmits signals across a much wider frequency than conventional radar systems (CW, FMCW, and SFCW). The advantages of UWB radar include high-resolution range measurement, high penetrability, multi-target detection, low susceptibility to atmospheric conditions, detection of barely discernible vital signs, and low power transmission [30,31,32]. As a result, UWB radar technology is widely used for through-wall detection. In addition, UWB radar with S-band frequency (2–4 GHz) can penetrate non-metal solid objects and detect human respiration behind the obstacle [33]. Specifically, UWB radar technology holds promising potential for human detection buried underneath the rubble in the wake of an earthquake.

A field programmable gate array (FPGA) is an integrated circuit which is configurable by a system designer ex post. The advantages of FPGA are high-speed and parallel processing, reconfigurability, and design flexibility [34,35,36,37]. In [38,39], FPGA was deployed to process received signals of through-wall radar systems.

In [40,41,42], integrated circuits (IC) based on complementary metal oxide semiconductor (CMOS) technology were proposed for UWB pulse generation, but the CMOS-based IC suffer from restricted UWB bandwidth. In [43], FPGA was used to generate UWB pulse with a center frequency of 110.25 MHz and a bandwidth of 41.5 MHz (fractional bandwidth of 0.376), rendering it unsuitable for through-wall detection. Based on [43], FPGA was used to generate IR-UWB pulse to improve transmitting frequency bandwidth for radar application, and the proposed FPGA scheme could achieve a bandwidth of 1.6 GHz (fractional bandwidth of 1.033) [44]. In [45], the FPGA scheme was further developed to detect live human subjects behind the wall by arm swing motion. The scheme achieved a bandwidth of 3.83 GHz (fractional bandwidth of 2) and could estimate the range of human subjects by using statistical standard deviation (SD). Table 1 compares previous works on IR-UWB technology and this current research.

This research thus proposes an FPGA scheme to generate IR-UWB pulse to detect from respiration live humans underneath the rubble and to estimate the range in order to locate the human subjects buried underneath. The FPGA scheme consists of three parts: digital clock manager, four-delay-paths stratagem, and edge combiner. Discrete fast Fourier transform is utilized to detect the respiration in terms of doppler frequency, and statistical 7th moment is used to locate human subjects in the range estimation. The proposed IR-UWB radar system is experimented with human subjects lying underneath different layers of stacked clay bricks in supine and prone positions.

The organization of this research is as follows: Section 1 is the introduction. Section 2 describes the proposed FPGA scheme and IR-UWB pulse generation. Section 3 details the human vital sign detection and range estimation algorithms. Section 4 deals with the experimental setup and method, and Section 5 discusses the experimental results. The concluding remarks are provided in Section 6.

## 2. IR-UWB Pulse Generation and FPGA Scheme

This section describes the ultra-wideband (UWB) pulse mathematical model and the proposed field programmable gate array (FPGA) scheme to generate an impulse radio ultra-wideband (IR-UWB) pulse.

### 2.1. UWB Pulse Mathematical Model 

The UWB pulse signal is mathematically characterized by Gaussian distribution function, which is expressed in Equation (1) [46]:

(1) $$G\left(t\right)=A_{0}e^{-\frac{1}{2}{\left(\frac{t}{a}\right)}^{2}}$$

where *A*_0_ is the transmission signal amplitude and *a* = *τ/√2* where *τ* is UWB pulse width.

**Figure 1:**
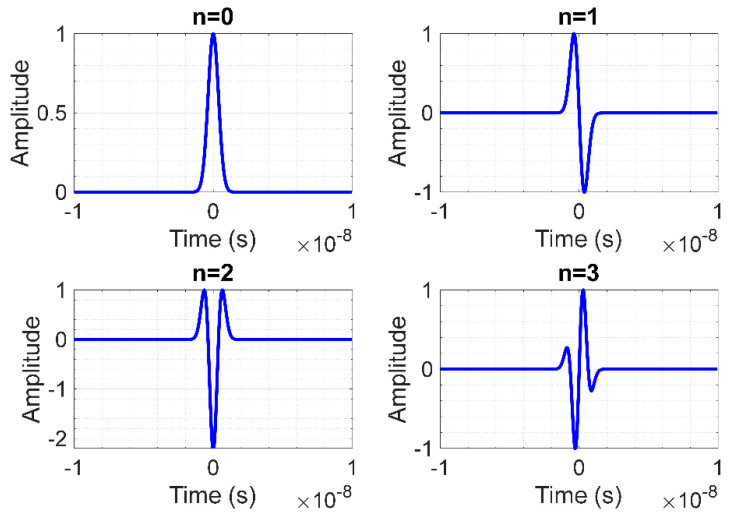
UWB pulse signals of 0th, 1st, 2nd, and 3rd order differential Gaussian distribution function.

The multi-order differential Gaussian distribution function is expressed in Equation (2), where *H_n_*(*t*) is Hermite polynomials that describe differential function (Equation (3)) and *n* is differential order (*n* = 0, 1, 2, 3, …) [46,47]. Figure 1 illustrates the 0th, 1st, 2nd, and 3rd order UWB pulse signals using Equation (2):

(2) $$G_{n}\left(t\right)=\frac{d^{n}}{dt^{n}}G\left(t\right)={\left(-1\right)}^{n}\frac{1}{a^{n}}H_{n}\left(t\right)G\left(t\right)$$

(3) $$H_{n}\left(t\right)={\left(-1\right)}^{n}\frac{1}{a^{n}}e^{\frac{1}{2}{\left(\frac{t}{a}\right)}^{2}}\frac{d^{n}}{dt^{n}}e^{-\frac{1}{2}{\left(\frac{t}{a}\right)}^{2}}$$

Specifically, this research proposes a field programmable gate array (FPGA) scheme to generate impulse radio ultra-wideband (IR-UWB) pulse whose shape resembles the 0th order differential Gaussian distribution function (*n* = 0). The IR-UWB pulse signal, given *n* = 0, is mathematically expressed in Equation (4) [48]:

(4) $$s\left(t\right)=A_{0}e^{-{\left(\frac{t}{\tau }\right)}^{2}}$$

### 2.2. Field Programmable Gate Array Scheme for IR-UWB Pulse Generation

In [43], an FPGA scheme was experimented to generate a UWB pulse. The scheme could achieve a fractional bandwidth of 0.376, with 110.25 MHz center frequency, 41.5 MHz bandwidth. Such a scheme also requires FPGA with tri-state buffers (TBUF). On the other hand, this current research proposes an FPGA scheme for generation of IR-UWB pulse in gigahertz (GHz) frequency band for detection of live human subjects buried underneath the rubble in the aftermath of an earthquake. Unlike in [43], the proposed scheme utilizes generic FPGA to generate IR-UWB pulse (i.e., Virtex-6 FPGA ML605).

**Figure 2:**
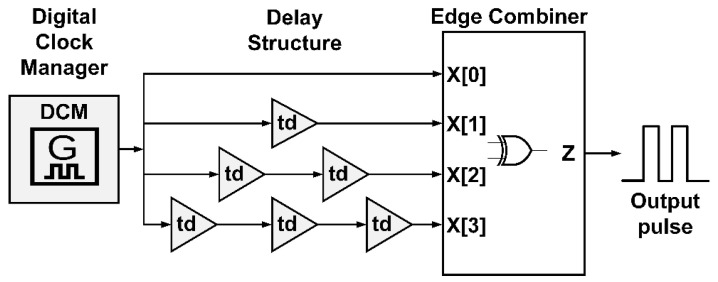
The proposed field programmable gate array (FPGA) scheme for IR-UWB pulse generation.

The proposed IR-UWB FPGA scheme consists of three parts: digital clock manager, delay path stratagem, and edge combiner. Figure 2 illustrates the proposed FPGA scheme for IR-UWB pulse generation, where *td* is delay time of individual buffer logic gates in FPGA architecture. 

#### 2.2.1. Digital Clock Manager

In the proposed scheme, the digital clock manager (DCM) in the FPGA intellectual property (IP) core is utilized to manipulate a low-voltage differential signaling (LVDS) oscillator. The LVDS oscillator is an on-board soldered clock source, resulting in highly reliable clock signal. 

**Figure 3:**
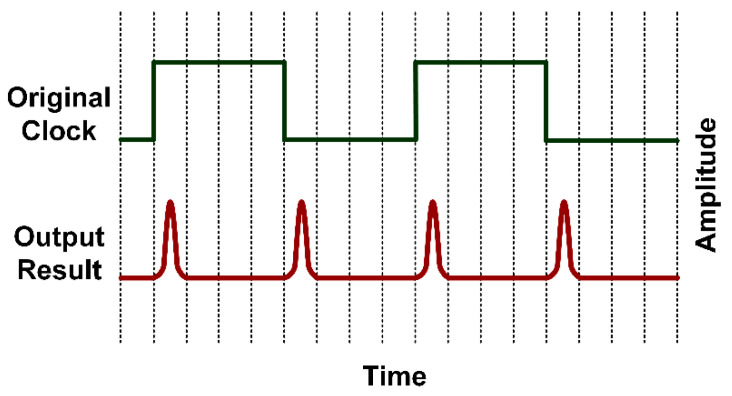
Relationship between pulse repetition frequency and initial clock frequency of low-voltage differential signaling (LVDS) oscillator.

More specifically, the DCM manipulates the initial clock frequency of the LVDS oscillator to realize predetermined pulse repetition frequency (PRF). In this scheme, the PRF is twice the LVDS initial clock frequency. The initial clock frequency of the LVDS oscillator of Virtex-6 FPGA ML605 is 5 MHz–200 MHz, giving rise to a wider range of PRF compared to [43]. Figure 3 shows the relationship between PRF and the initial clock frequency of LVDS oscillator. 

#### 2.2.2. Delay Path Stratagem

**Figure 4:**
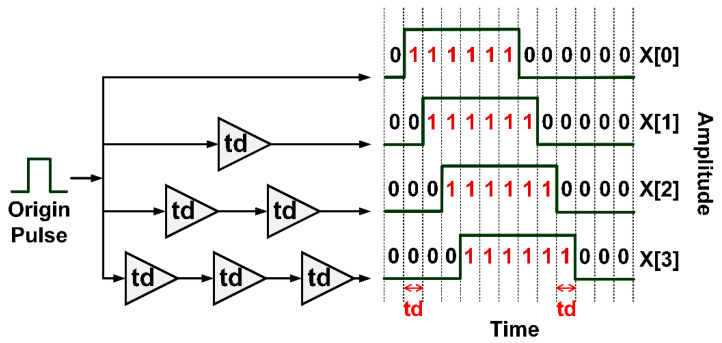
The staggered time-delay stratagem with four delay paths.

In FPGA, time-delay paths are predetermined by configuring the sequence of buffer logic gates. In this research, the delay path stratagem consists of four time-delay paths: X[0], X[1], X[2], and X[3], given 40 nm Virtex-6 FPGA ML605 [49]. The first delay path (X[0]) contains no delay time (*td*) and the second, third, and fourth delay paths (X[1], X[2], X[3]) have one, two, and three units of delay time, thereby resulting in staggered time-delay paths (Figure 4). To generate an IR-UWB pulse and achieve very broad bandwidth, a minimum delay time (*td*) is required.

In order to realize the minimum delay time, the delay times of buffer logic gates in FPGA architecture are determined from the look up table (LUT) and the path with the minimum delay time is configurated by using a configuration logic block (CLB) based on the user constraint file. The place and route (P&R) algorithm is implemented to route the configured buffer logic gates. The routing between buffer logic gates is validated by the Xilinx PlanAhead Design and Analysis Tool [50]. Figure 4 depicts the proposed staggered time-delay stratagem with four time-delay paths.

#### 2.2.3. Edge Combiner

In the edge combiner part, exclusive OR (XOR) is applied to four staggered delay paths to achieve four narrow active pulses per cycle, where one active pulse is equal to one unit of delay time (*td*). LUT algorithm is utilized and functions as concurrent four-input XOR gate. The number of inputs is identical to the four time-delay paths in the delay path stratagem: X[0], X[1], X[2], and X[3]. The output of the edge combiner (z) is fed into onboard coupling capacitor connected with 50Ω SMA connector to generate IR-UWB pulse (o) whose shape resembles the 0th order differential Gaussian distribution function (*n* = 0). The IR-UWB pulse width is approximately twice the delay time (*td*).

**Figure 5:**
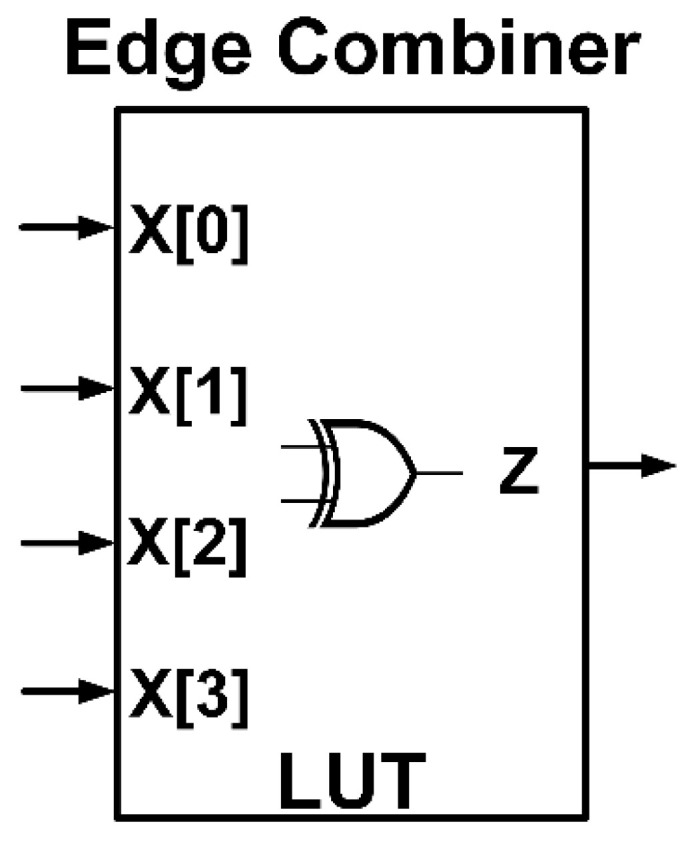
Edge combiner with look up table (LUT) algorithm as concurrent four-input XOR gate.

**Figure 6:**
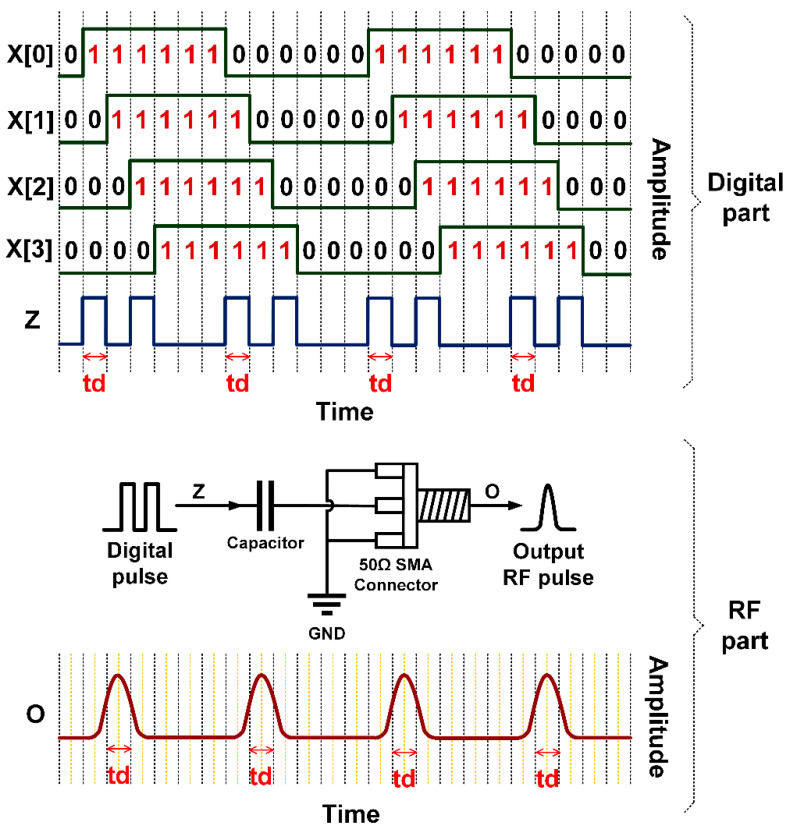
Application of XOR to four staggered delay paths (X[0], X[1], X[2], and X[3]) for four narrow active pulses per cycle where *z* is the output of edge combiner.

The advantages of the LUT algorithm are shorter processing time and lower time delay effect, vis-à-vis non-LUT edge combiner. Figure 5 illustrates the edge combiner with LUT algorithm as concurrent four-input XOR gate. Figure 6 shows the four staggered delay paths and four narrow active pulses per cycle, where z is the output of edge combiner.

## 3. Human Detection and Range Estimation Algorithms

### 3.1. Vital Sign Model Underneath the Rubble

In theory, electromagnetic (EM) waves which are reflected off an object scatter and/or are captured by a receiver [51,52,53,54,55,56]. The back-scattered signal is mathematically written in Equation (5).

(5) $$R\left(t,\tau \right)=\underset{p}{\sum }\sigma_{p}s\left(t-t_{p}\right)+\underset{o}{\sum }\sigma_{o}s\left(t-t_{o}\left(\tau \right)\right)+\underset{v}{\sum }\sigma_{v}s\left(t-t_{v}\left(\tau \right)\right)$$

where *σ_p_*, *σ_o_*, *σ_v_* are stationary, non-stationary, and human objects, *s*(*t* − *t_v_*) is time-shifting of the transmitted signal, *t* and *τ* are fast-time and slow-time domains.

**Figure 7:**
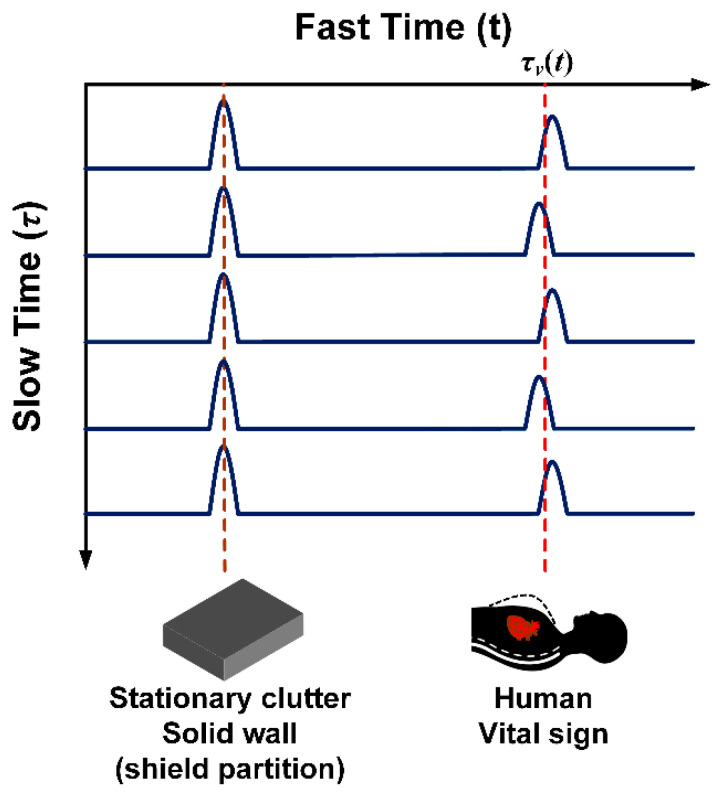
Reflected signals from human and solid object in fast time and slow time.

Figure 7 illustrates the reflected signals from the human subject and impenetrable solid object in fast time and slow time. The reflected signals from the human subject are periodic due to the vital signs, i.e., respiration and heartbeat. Meanwhile, the reflected signals from the solid object are static as they are not subject to time shift. As a result, human physiological movements can be determined by time shift of reflected signals.

Since this research focuses on human vital signs (i.e., reflected signals from human), the back-scattered signal in Equation (5) can be simplified as:

(6) $$R\left(t,\tau \right)=\sigma_{v}s\left(t-t_{v}\left(\tau \right)\right)$$

where the time delay (*t_v_*(*τ*)) is equal to 2*d*(*τ*)/*c*, where *c* is the speed of light (3 × 10^8^ m/s). The distance between the transmitting (*Tx*) antenna and human subject (d(τ)) is estimated by:

(7) $$d\left(\tau \right)=d_{0}+d_{h}\sin\left(2\pi f_{h}\tau \right)+d_{r}\sin\left(2\pi f_{r}\tau \right)$$

where *d*_0_ is the nominal distance between the Tx antenna and human subject, *d_h_* and *d_r_* are the movement amplitudes of the heart and respiration, *f_h_* and *f_r_* are the frequencies corresponding to heartbeat and respiration. From Equation (7), the time delay function can be written in Equation (8):

(8) $$t_{v}\left(\tau \right)=2d\left(\tau \right)/c=t_{0}+t_{h}\sin\left(2\pi f_{h}\tau \right)+t_{r}\sin\left(2\pi f_{r}\tau \right)$$

where *t*_0_, *t_r_*, and *t_h_* are the delays related to the human distance, respiratory and heart motions, respectively.

**Figure 8:**
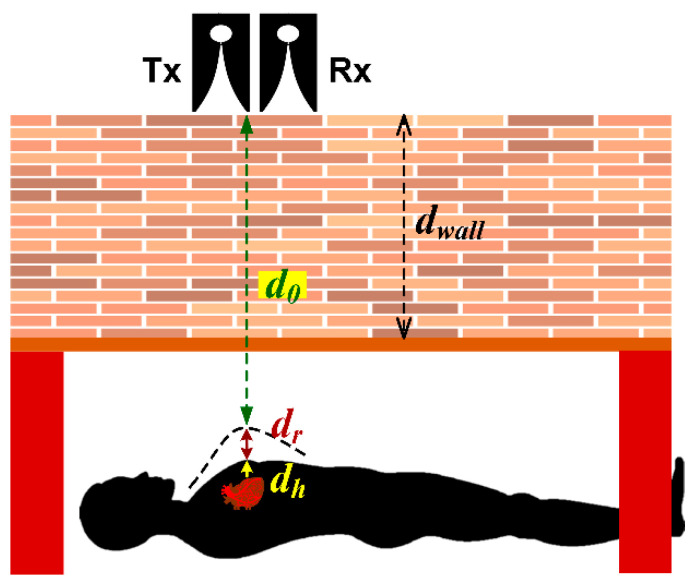
The distance between antenna and human subject as a function of the movement amplitudes of the heart and respiration.

Figure 8 illustrates the distance between antenna and human subject ($d_{0}$) as a function of the movement amplitudes of the heart ($d_{h}$) and respiration ($d_{r}$). The proposed through-obstacle IR-UWB radar is of classical monostatic radar, consisting of one Vivaldi transmitting (1-Tx) and one Vivaldi receiving (1-Rx) antenna.

Due to challenges in measuring the back-scattered signal in continuous time (Equation (5)), this research characterizes the back-scattered signal in discrete time [51,52,53,54,55,56,57]. The back-scattered signal in discrete time (*R*[*k*,*l*]) is expressed in Equation (9):

(9) R[k,l]=h[k,l]+c[k,l]+w[k,l]+q[k,l]

where *h*[*k*,*l*], *c*[*k*,*l*], *w*[*k*,*l*], and *q*[*k*,*l*] are the respiration and heartbeat signals, static signal, white noise, and non-static signal, respectively, where *k* is discrete domain in fast time of *K* sampling and *l* is discrete domain in slow time of *L* sampling. Preprocessing is subsequently performed to filter out unwanted signals (i.e., static signal, white noise, and non-static signal) to extract the vital signs.

### 3.2. Preprocessing To Remove Unwanted Signals

The back-scattered signal in discrete time is preprocessed to remove unwanted signals. The unwanted static signal (*c*[*k*,*l*]) is independent of slow time (Figure 7) and can be removed by averaging *l* by the number of slow time sampling *(L*). The discrete-time back-scattered signal with the unwanted static signal (*c*[*k*,*l*]) removed is expressed in Equation (10):

(10) $$\Omega_{n}\left[k,l\right]=R\left[k,l\right]-\frac{1}{L}\overset{L}{\underset{l=1}{\sum }}R\left[k,l\right]=h\left[k,l\right]+w\left[k,l\right]+q\left[k,l\right]$$

In practice, the first and second pulses of back-scattered signal are the antenna coupling and wall/obstacle reflection, respectively. Given the antenna coupling and wall reflection at the minimum detectable range, this research designates the first and second pulses as zero:

(11) $$\Omega \left[1:K_{zero},\;l\right]=0,\;\mathrm{where}\;\;K_{zero}=\mathrm{floor}d_{min}/\Delta d$$

where *d_min_* is the minimum detectable range and Δd is the range resolution of radar depending on the speed of light (*c*) and bandwidth (*B*) where Δd = c/2B.

The non-static signal (*q*[*k*,*l*]) and white noise (*w*[*k*,*l*]) are reduced by smooth filter (Equation (12)) and Butterworth bandpass filter (Equation (13)) in fast and slow-time domains:

(12) $$y\left[k,l\right]=\frac{1}{2\lambda +1}\overset{\lambda }{\underset{i=-\lambda }{\sum }}\Omega \left[k-i,l\right]$$

where *λ* is the number of average data points on either side of Ω[*k*,*l*] and 2λ + 1 is the span. The transfer function of Butterworth bandpass filter is expressed as:

(13) $${\left|H\left(\omega \right)\right|}^{2}=\frac{1}{1+{\left(\omega /\omega_{c}\right)}^{2N_{f}}}$$

where *ω_c_* is the cutoff frequency and *N_f_* is the filter order and set to 5, giving a good tradeoff between performance and complexity [54]. The discrete-time back-scattered signal with unwanted signals removed is rewritten as:

(14) $$y\left[k,l\right]\approx h\left[k,l\right]+w_{0}\left[k,l\right]+q_{0}\left[k,l\right]\;\;\;\;\;\;$$

### 3.3. Respiratory Rate and Range Estimation

The discrete fast Fourier transform (DFT) algorithm is applied to the discrete-time back-scattered signal in Equation (14) to determine doppler frequency (i.e., respiratory rate). The DFT of the refined discrete-time back-scattered signal is expressed in Equation (15).

(15) $$Y\left[k,f\right]=\overset{L}{\underset{l=1}{\sum }}y\left[k,l\right]e^{-\frac{j2\pi fl}{L}}$$

**Figure 9:**
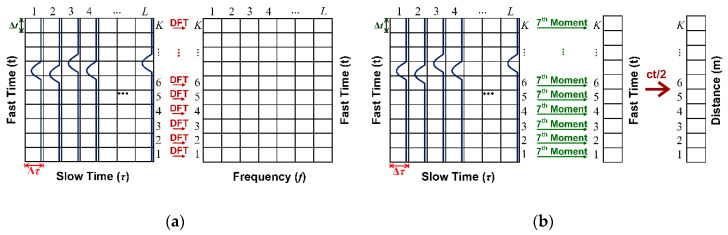
Respiration rate and range estimation: (**a**) DFT doppler frequency; (**b**) statistical 7th moment.

In range estimation, the statistical 7th moment is used to locate human subjects underneath the rubble. From Equation (14), the statistical 7th moment can be expressed in Equation (16), where Equation (17) is the mean of y[k,l] in slow time. Figure 9a,b respectively illustrates the DFT doppler frequency (respiration rate) and range estimation using the statistical 7th moment.

(16) $$\mu_{7}=\frac{1}{L}\overset{L}{\underset{l=1}{\sum }}{\left(y\left[k,l\right]-\bar{y}\left[k\right]\right)}^{7}$$

(17) $$\bar{y}\left[k\right]=\frac{1}{L}\overset{L}{\underset{l=1}{\sum }}y\left[k,l\right]$$

## 4. Experimental Setup and Method

This section deals with the generation of IR-UWB pulse using the proposed FPGA scheme and with detection of human subjects underneath the rubble using the FPGA scheme.

### 4.1. IR-UWB Pulse Generation

**Figure 10:**
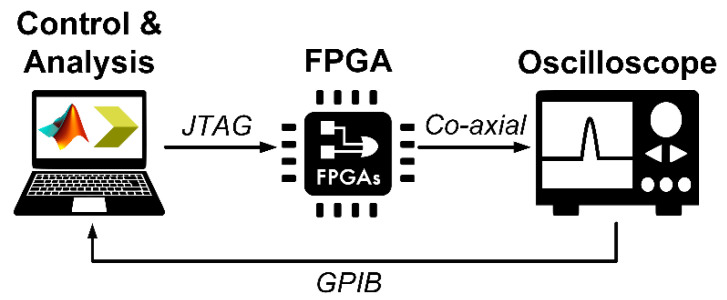
Schematic of IR-UWB pulse generation using the proposed FPGA scheme.

Figure 10 illustrates the schematic of IR-UWB pulse generation using the proposed FPGA scheme. The Verilog HDL programming language is used to create a user constraint file for selectively placing and routing buffer logic gates; and to regulate LVDS clock at 10 MHz. An FPGA bitstream file (.bit) is compiled and programmed into FPGA via Joint Test Action Group (JTAG) connector. The setup is verified by embedded hardware monitoring function ChipScope Pro Analyzer.

The IR-UWB pulse from FPGA is fed into oscilloscope via co-axial transmission line. A computer is used to retrieve digital data via general purpose interface bus (GPIB) for MATLAB analysis to determine the pulse duration and bandwidth. Table 2 tabulates the experimental components and specifications.

### 4.2. Detection of Human Underneath the Rubble

**Figure 11:**
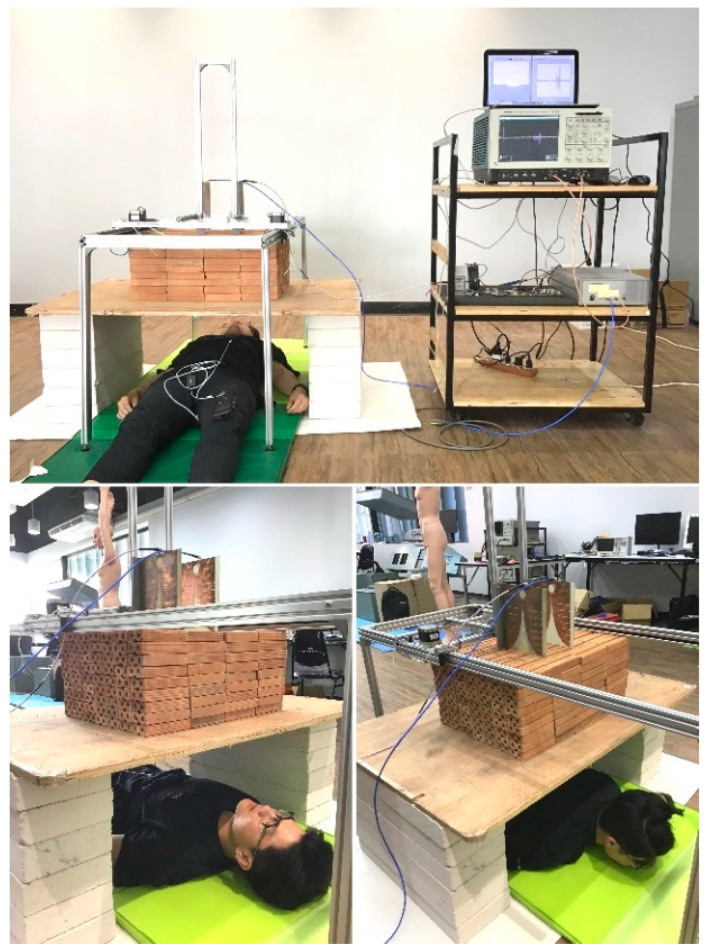
Experimental setup for detection of human subject underneath layers of stacked clay bricks in supine and prone positions using the FPGA scheme.

To simulate detection of human subjects buried underneath the rubble, this research used layers of stacked clay bricks as a substitute for collapsed walls. Figure 11 depicts the experimental setup for detection of human subjects underneath layers of stacked clay bricks in supine and prone positions using the FPGA scheme. The human participants were instructed to remain physically stationary over the course of experiment.

In the experimental detection, there were three male and three female healthy participants. The human subjects individually lay underneath layers of stacked clay bricks with Tx and Rx Vivaldi antennas sitting atop. The distance between Tx and Rx antennas is 15 cm, and the radar-antennas set is motionable horizontally and vertically.

The layers of stacked clay bricks on 1.5 cm-thick plywood were varied between 3, 6, and 9 layers (9, 18, and 27 cm in thickness). The distances between the Tx-Rx antennas and participating human subjects underneath the stacked clay bricks were 20.5–47 cm, depending on the number of brick layers (3, 6, and 9 layers) and gender. The vital sign detection experiments were carried out with participants in supine and prone position. The data acquisition duration for each individual participant in either position was 3 min with 256 slow-time pulses and 5000 fast-time datapoints.

**Figure 12:**
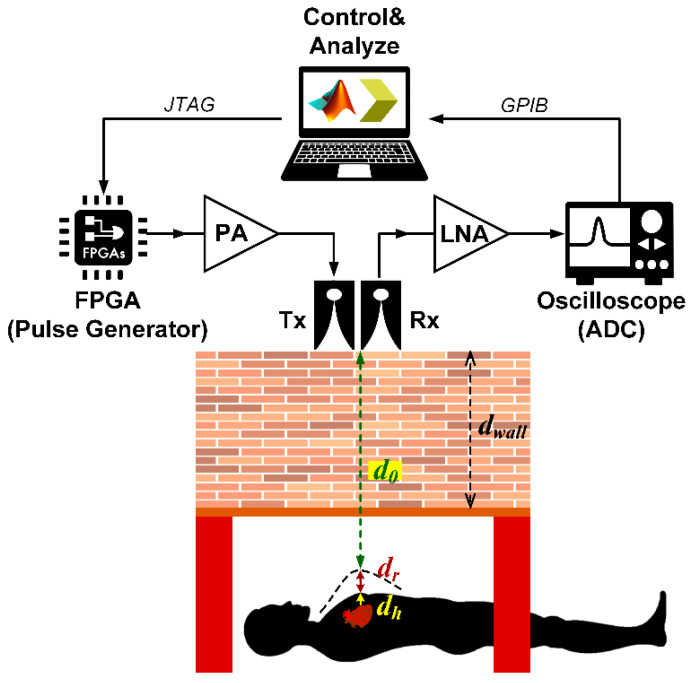
Schematic of IR-UWB pulse generation using FPGA scheme for detection of human subject underneath layers of stacked clay bricks.

In addition, the six participants wore a respiration sensor (BioRadio^TM^ respiratory kit) around their chest to monitor respiration rhythm. The experimental measurement using the proposed IR-UWB FPGA scheme was validated against the readings by the respiration sensor. Figure 12 shows the schematic of IR-UWB pulse generation using FPGA scheme for detection of human subjects underneath layers of stacked clay bricks. The specifications of the experimental components and equipment are presented in Table 2.

## 5. Experimental Results and Discussion

This section discusses IR-UWB pulse generated by the proposed FPGA scheme and the human detection performance of the IR-UWB radar system in terms of respiratory rate and range estimation.

### 5.1. IR-UWB Pulse Generation Using FPGA Scheme

**Figure 13:**
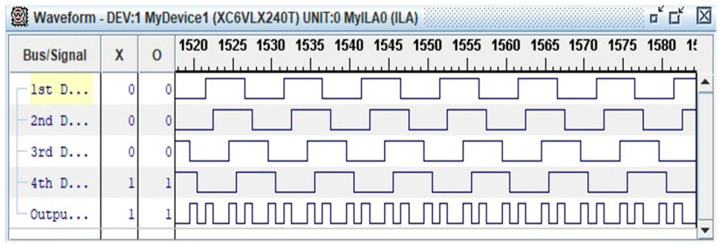
Waveform from ChipScope Pro Analyzer function.

Figure 13 illustrates the waveform of LUT four-input XOR gate by using ChipScope Pro Analyzer function. The waveform is identical to the schematic of four staggered delay paths and four narrow active pulses or delay time (*td*) per cycle (Figure 6). One unit of delay time (*td*) is approximately 270 ps.

**Figure 14:**
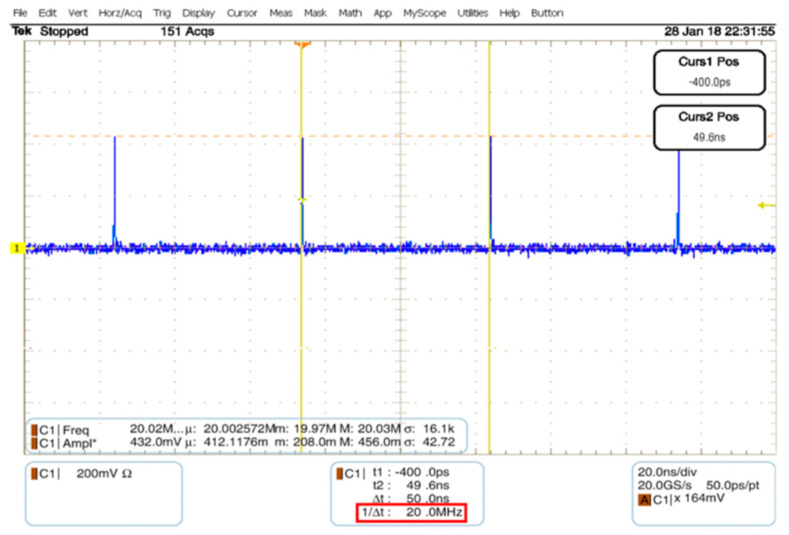
IR-UWB pulse generated by the FPGA scheme with pulse repetition frequency of 20 MHz using digital oscilloscope.

**Figure 15:**
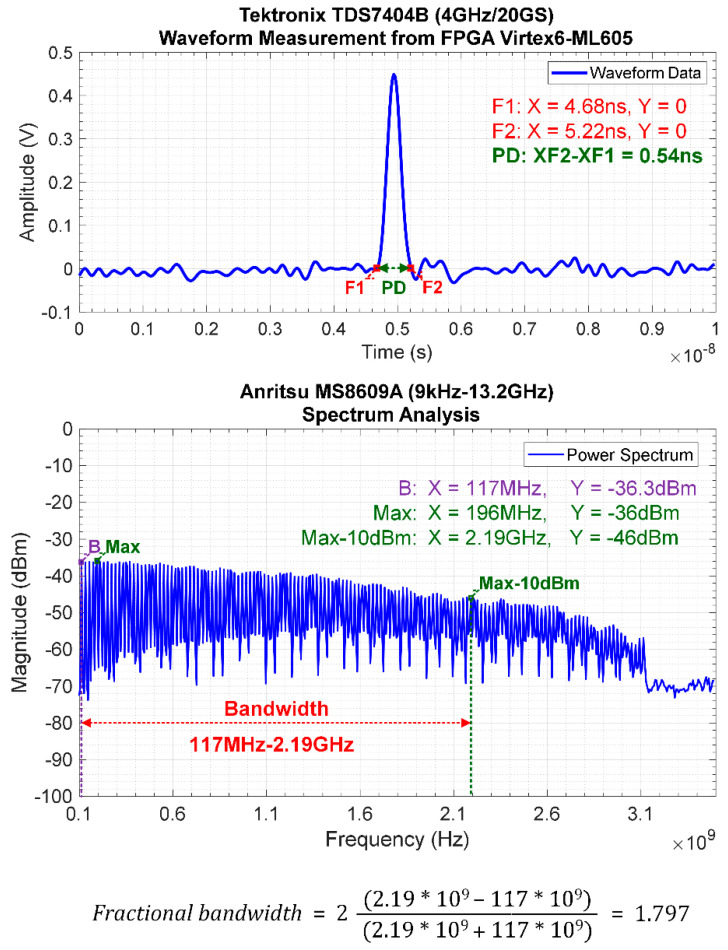
Pulse duration and bandwidth of IR-UWB generated by the FPGA scheme.

Figure 14 depicts the IR-UWB pulse generated by the FPGA scheme with pulse repetition frequency (PRF) of 20 MHz using oscilloscope. Given the LVDS clock of 10 MHz, the PRF of IR-UWB is 20 MHz, which is twice the LVDS clock frequency. The digital data from the oscilloscope and spectrum analyzer were retrieved via GPIB to characterize the pulse duration and bandwidth using MATLAB, as shown in Figure 15. The pulse duration is 540 ps, which is approximately twice the delay time (*td*), and the bandwidth is 2.073 GHz (117 MHz–2.19 GHz, fractional bandwidth of 1.797), given the Federal Communications Commission (FCC)’s normalized magnitude ≥ −10 dB. The realized IR-UWB bandwidth falls within the S-band frequency (2–4 GHz).

### 5.2. Human Detection Performance of IR-UWB Radar System

This sub-section discusses the performance of the proposed IR-UWB radar system to detect human respiration and locate the human subjects underneath the rubble (range estimation). As previously stated, this research used layers of stacked clay bricks to simulate detection of human subjects buried underneath the rubble. Since the detection results of the six participating human subjects are insignificantly different, this part thus presents the findings of one single participant.

#### 5.2.1. Respiratory Rate Estimation

**Figure 16:**
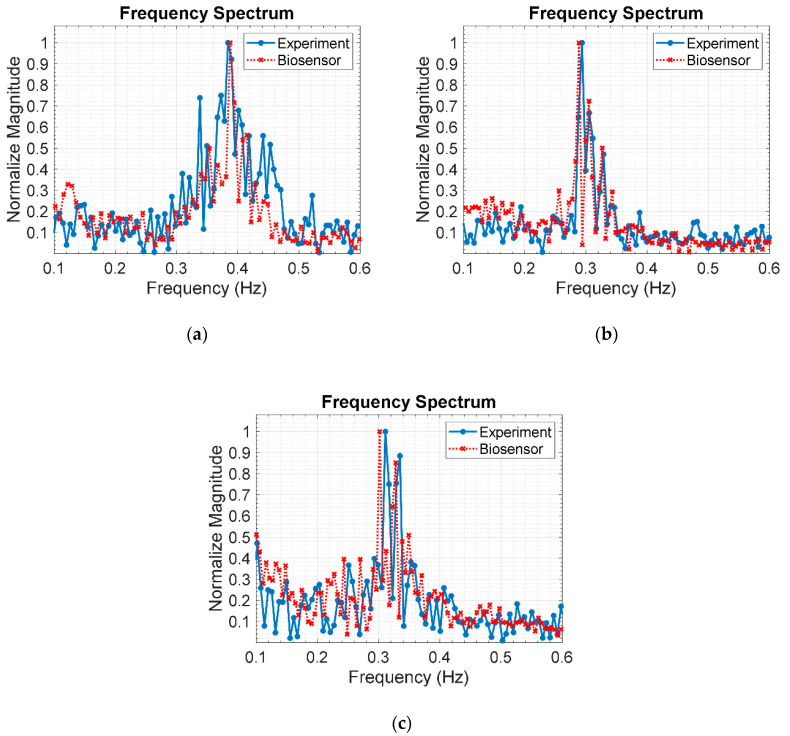
Respiratory frequency of a human subject in supine position under layers of stacked clay bricks, using IR-UWB radar system and respiration sensor: (**a**) 3 layers (9 cm); (**b**) 6 layers (18 cm); (**c**) 9 layers (27 cm).

Figure 16a–c respectively illustrates the respiratory frequency of a human subject in supine position under layers of stacked clay bricks: 3 (9 cm), 6 (18 cm), and 9 layers (27 cm), using the IR-UWB radar system and respiration sensor. The experimental respiratory frequency, as indicated by the maximum frequency peak, are 0.3842 Hz (23 breaths/min), 0.2938 Hz (17.6 breaths/min), and 0.3111 Hz (18.7 breaths/min) for 3, 6, and 9 layers of stacked bricks. The corresponding benchmark respiratory frequency, as measured by the respiration sensor, are 0.3878 Hz (23.3 breaths/min), 0.2888 Hz (17.3 breaths/min), and 0.3017 Hz (18.1 breaths/min). The experimental and benchmark respiratory rates are in good agreement, suggesting that the proposed IR-UWB radar system is applicable to localization of human buried underneath the rubble.

**Figure 17:**
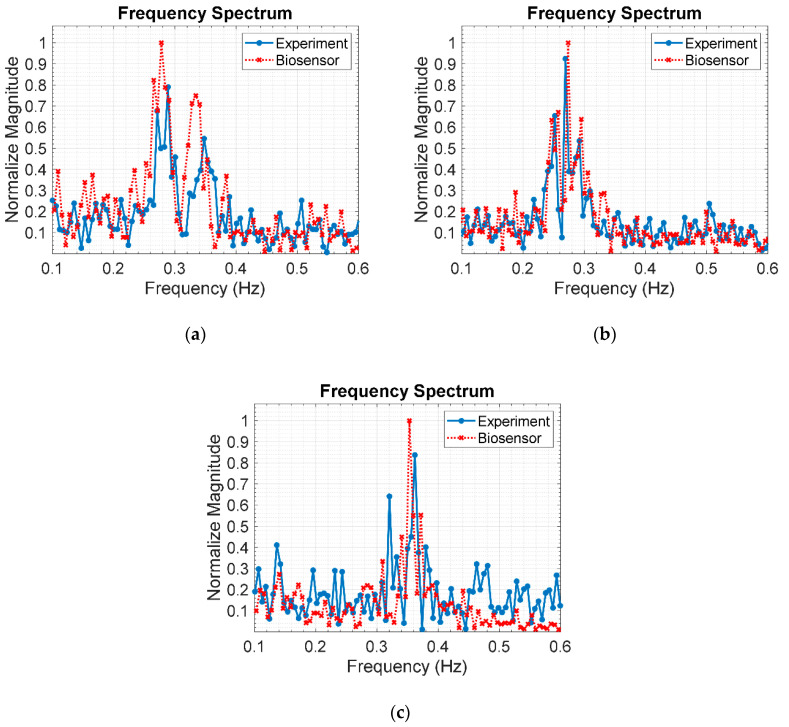
Respiratory frequency of a human subject in prone position under layers of stacked clay bricks, using IR-UWB radar system and respiration sensor: (**a**) 3 layers (9 cm); (**b**) 6 layers (18 cm); (**c**) 9 layers (27 cm).

Figure 17a–c respectively depicts the respiratory frequency of the human subject in the prone position under different thicknesses of clay bricks: 9 (3 layers), 18 (6 layers), and 27 cm (9 layers), using the IR-UWB radar system and respiration sensor. The experimental respiratory frequencies are 0.2890 Hz (17.3 breaths/min), 0.2694 Hz (16.2 breaths/min), and 0.3621 Hz (21.7 breaths/min) for 3, 6, and 9 layers of stacked bricks. The corresponding benchmark respiratory frequency, as measured by the respiration sensor, are 0.2781 Hz (16.7 breaths/min), 0.2738 Hz (16.4 breaths/min), and 0.3531 Hz (21.2 breaths/min). The experimental and benchmark respiratory rates are in good agreement, indicating that the IR-UWB radar system is applicable to human localization underneath the rubble.

#### 5.2.2. Range Estimation Based on Doppler Frequency

**Figure 18:**
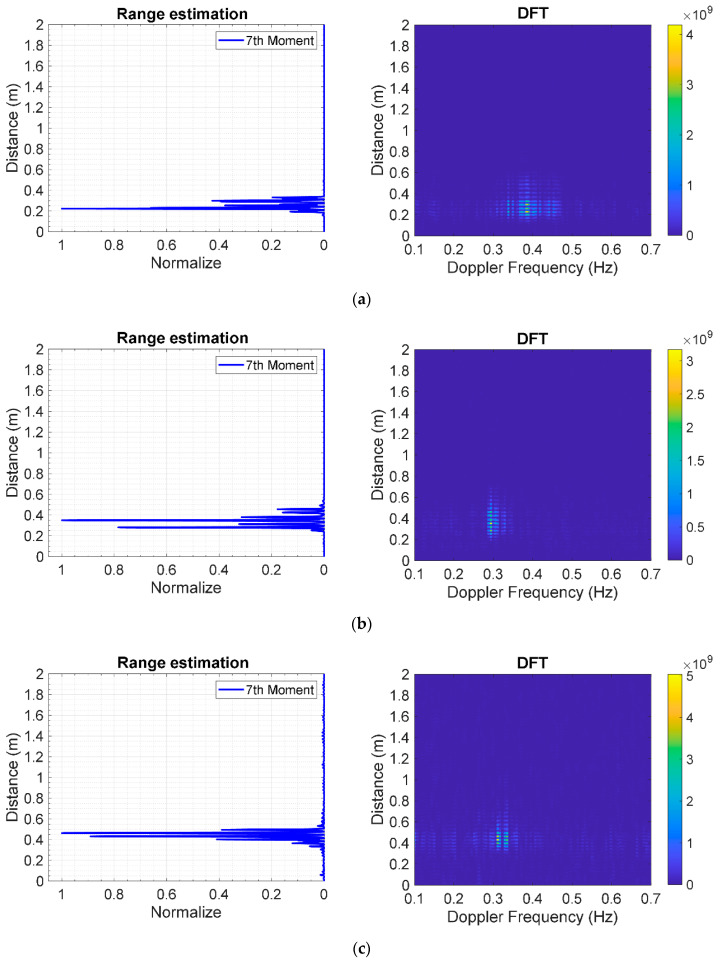
Statistical 7th-moment range estimation of a human subject in supine position under layers of stacked clay bricks using IR-UWB radar system: (**a**) 3 layers (9 cm); (**b**) 6 layers (18 cm); (**c**) 9 layers (27 cm).

Figure 18a–c respectively shows the estimated range of the human subject in supine position under different thicknesses of stacked clay bricks: 9 (3 layers), 18 (6 layers), and 27 cm (9 layers). In range estimation, the statistical 7th moment is used to locate human subjects underneath the rubble. The estimated distances (range) between the radar antennas and the human subject are 22.33, 35.08, and 46.32 cm for 3, 6, and 9 layers of stacked bricks. Meanwhile, the corresponding distances, as measured by laser measuring device, are 24.5, 33.5, and 42.5 cm. The experimental and measured results are in good agreement, indicating the applicability of the IR-UWB radar system for human localization underneath the rubble.

**Figure 19:**
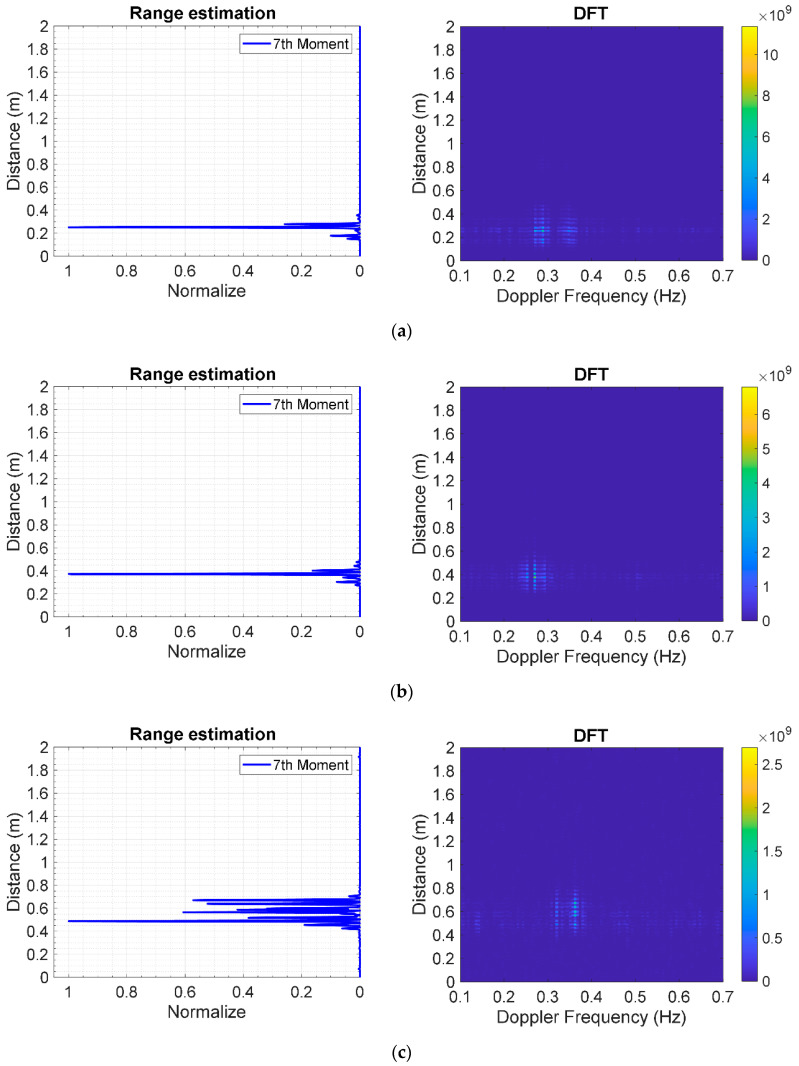
Statistical 7th-moment range estimation of a human subject in prone position under layers of stacked clay bricks using IR-UWB radar system: (**a**) 3 layers (9 cm); (**b**) 6 layers (18 cm); (**c**) 9 layers (27 cm).

Figure 19a–c respectively illustrate the estimated range of the human subject in prone position under different thicknesses of clay bricks: 9 (3 layers), 18 (6 layers), and 27 cm (9 layers). The estimated distances between the antennas and human subject are 25.18, 37.32, and 48.72 cm for 3, 6, and 9 layers of stacked bricks. The corresponding distances, as measured by laser measuring device, are 25.5, 34.5, and 43.5 cm. The experimental and measured results are in good agreement. As illustrated in Figure 18 and Figure 19, statistical 7th moment improves the range estimation performance of the IR-UWB radar system, vis-à-vis DFT.

## 6. Conclusions

This research proposes an FPGA scheme to generate an IR-UWB pulse based on four time-delay paths using LUT four-input XOR gate. The FPGA scheme consists of three parts: digital clock manager, delay path stratagem, and edge combiner. The IR-UWB radar system is designed to detect from respiration live humans buried underneath the rubble in the wake of an earthquake. The IR-UWB radar system is also capable of estimating the range to locate human subjects buried underneath. Discrete fast Fourier transform is utilized to detect the respiration in terms of Doppler frequency, and the statistical 7th moment is used to locate human subjects underneath the rubble. Experiments were carried out with human subjects lying underneath different layers of stacked clay bricks in supine and prone position. The results show that the IR-UWB radar system achieves a pulse duration of 540 ps and a bandwidth of 2.073 GHz (117 MHz–2.19 GHz, fractional bandwidth of 1.797). In respiration detection, the experimental results are in good agreement with the respiration sensor readings, indicating that the IR-UWB radar system is capable of human detection underneath the rubble. In addition, the estimated ranges of the human location buried underneath using the IR-UWB radar system are agreeable with those measured by a laser measuring device. In essence, the proposed technology could be further improved for rescue operation in the aftermath of an earthquake.

## Figures and Tables

**Table 1:** Comparison between previous works on impulse-radio ultra-wideband (IR-UWB) technology and this current research.

Specifics	Ref. [44]	Ref. [45]	Current Research
IR-UWB bandwidth	1.6 GHz	3.83 GHz	2.19 GHz
Fractional bandwidth	1.033	2	1.797
Pulse repetition frequency (PRF)	200 MHz, 400 MHz	20 MHz	20 MHz
Signal amplitude	611 mV	432 mV	456 mV
Human detection method	N/A	Arm swing motion (macro-doppler)	Vital sign (respiration) (micro-doppler)
Range estimation method	N/A	Standard Deviation	7th moment
Range validation	N/A	Yes	Yes
Macro/Micro doppler validation	N/A	No	Yes (Respiration sensor)

**Table 2:** The specifications of experimental components.

Components	Model Name	Specification
Oscilloscope (ADC)	Tektronix, TDS7404B	Digital Phosphor Oscilloscope (4 GHz, 20 GS/s)
FPGA	Xilinx, Virtex 6-ML605 (XC6VLX240T)	Total logic cell: 241,152 cells Technology process: 40 nm Copper CMOS process
GPIB	Agilent Technologies, 82357B	USB/GPIB interface USB2.0, transfer rate over 850 KB/s
Power Amplifier (PA)	Mini Circuits, ZVE-8G	2 GHz–8 GHz, Gain = 30 dBm
Low Noise Amplifier (LNA)	R&K-AA260-0S	2 GHz–5 GHz, Gain = 26 dBm
Spectrum Analyzer	Anritsu, MS8609A	Digital Mobile Radio Transmitter Tester, 9 kHz–13.2 GHz
Tx, Rx Antennas	Vivaldi antenna	0.7 GHz–2.5 GHz, Gain = 11 dBi
Respiratory measurement sensor	Great Lakes Neurotechnologies, BioRadio^TM^	Wireless Biomedical monitor, Chest Interface cables belt sensor.
